# Breaking the rules of Rubisco catalysis

**DOI:** 10.1093/jxb/erw197

**Published:** 2016-05-28

**Authors:** David T. Hanson

**Affiliations:** Department of Biology University of New Mexico, Albuquerque, New Mexico 87131, USA

**Keywords:** Algae, carbon fixation, diatoms, kinetics, photosynthesis, Rubisco.

**The canon of knowledge on the catalytic properties of the photosynthetic enzyme Rubisco has shackled efforts to understand its diversity. Now the chains are off. While investigating the variability in Rubisco function among diatoms, Young *et al.* (see pages 3445–3456 in this issue) have demonstrated that it is our thinking, not Rubisco catalysis, that has been constrained.**

The photosynthetic CO_2_-fixing enzyme, ribulose-1,5-bisphosphate carboxylase/oxygenase (Rubisco), is renowned for its slow catalytic rate and difficulty in distinguishing between the substrate of photosynthesis, CO_2_, and one of the products, O_2_. The oxygenase activity was discovered 45 years ago (Bowes *et al.*, 1971), nearly 20 years after the discovery of the carboxylase activity (Quayle *et al.*, 1954; Weissbach *et al.*, 1954). The photorespiratory losses associated with Rubisco oxygenation are substantial (Sharkey, 1988), and the persistence of oxygenation throughout 3.5 billion years of evolution – particularly the last 400 million years when the O_2_ and CO_2_ pressures in the atmosphere have favored substantial oxygenation and photorespiration – is still a mystery (Hagemann *et al.* 2016).

It is conceivable that evolutionary constraints related to the origins of Rubisco from enzymes with roles in sulfur metabolism (Ashida *et al.*, 2003) may have imposed limitations on the catalytic mechanism for CO_2_ fixation, though none have come to light. Oxygenation has also been proposed as an energy dissipative mechanism that would be beneficial under some stressful conditions (Osmond, 1981), but this does not explain why it exists in all Rubiscos, including those from anaerobic organisms (Andrews and Lorimer, 1987).

The inefficiency of Rubisco is due not only to the strong competitive interaction of O_2_ and CO_2_ at the active site, but also to a slow catalytic turnover rate per active site (*k*_cat_) and a low affinity for CO_2_ (high *K*_C_). Substrate-saturated *k*_cat_ values (maximum CO_2_ fixation per catalytic site) occur in the range 1–12s^–1^ and the *k*_cat_/*K*_C_ ratios (which reflect the ability of Rubisco to function when CO_2_ is limiting) occur in the range 2–40×10^4^ M^–1^ s^–1^ (Badger *et al.*, 1998). This feeble catalytic potency at limiting CO_2_ is several orders of magnitude slower than the diffusion limit to catalysis that many enzymes approach (10^8^–10^9^ M^–1^ s^–1^) and it means that plants need copious amounts of Rubisco (as much as 50% of soluble leaf protein, consuming up to 25% of leaf nitrogen) to support adequate rates of photosynthesis in our current atmosphere (Andrews and Lorimer, 1987). The requirement for such large quantities of Rubisco gives it the dubious honor of being the most abundant protein on Earth (Ellis, 1979). Even with so much Rubisco present, it is still often the rate-limiting enzyme in photosynthesis.

## Target for manipulation

Scientists around the world who were interested in increasing plant productivity saw this suite of catalytic problems and recognized that it made Rubisco a good target for manipulation (Andrews and Lorimer, 1987; Parry *et al.*, 2013). However, even after the first 25 crystal structures were available for Rubiscos from eight divergent species (Form I Red and Green types, and Form II) along with a plethora of sequences, there was next to nothing that could explain observed variation in catalysis (Andersson and Taylor, 2003). Furthermore, efforts to genetically modify the enzyme were hampered by its complex structure and the difficulty of assembling functional enzymes in workhorse organisms like *Escherichia coli*. This has increased the need to study kinetic properties of Rubisco from a more diverse range of species (Parry *et al.*, 2013), while simultaneously convincing many that improvements are highly unlikely. The few surveys of a handful of catalytic properties showed some patterns of improvement from cyanobacteria to plants within the green lineage, along with trade-offs between increased *k*_cat_ and decreased selectivity of Rubisco in species that express a carbon-concentrating mechanism (CCM). This favoring of speed over affinity and selectivity works because CCMs elevate the level of CO_2_ near the active sites of Rubisco (Badger *et al.*, 1998). These observations led to the hypothesis that Rubisco was already optimized in photosynthetic organisms (Tcherkez *et al.*, 2006) and that the kinetics were constrained in a narrow, one-dimensional landscape (Savir *et al.* 2010).

The findings of Young *et al.* (2016) now challenge the generality of this hypothesis. The work describes the survey of Rubisco catalytic properties as a diagnostic tool for the enzymes’ subcellular environment in diatoms (Box 1), providing a proxy for examining CCM efficiency. They found a remarkable range of diversity in Rubisco kinetics within this Form I Red-type lineage, suggesting a wide range of cellular environments and CCM function. This in itself is an interesting and important finding since it has been challenging to study CCM function in diatoms. However, the astonishing discovery is that the canonical linear relationship between speed and affinity does not exist, for either CO_2_ or O_2_, in Rubisco from these species (Young *et al.*, 2016; Fig. 3A, D), and their data greatly weakens or eliminates the broader relationship when examined together with Rubiscos from other species.

**Box 1. Ancient phytoplankton**

Centric diatom frustules from ancient marine sediments at site 1090 in the Atlantic sector of the Southern Ocean (samples obtained from the Integrated Ocean Drilling Program at the Bremen Core Repository in Bremen, Germany). These diatoms are from the Eocene–Oligocene boundary (about 35 million years ago). SEM; scale bar = 10 μm. Courtesy of Dr Ana Heureux.

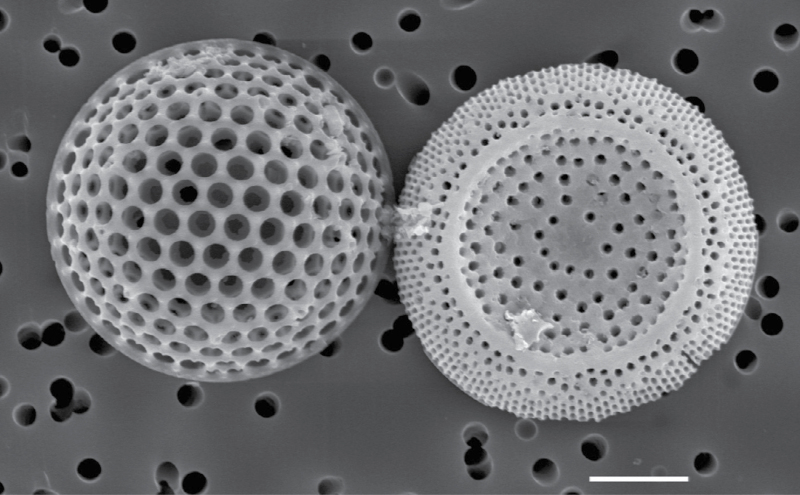

Some might dismiss the significance of their findings since the affinities are low for CO_2_, but they are also lower for O_2_ and to a different extent. However, it is not the direction of the variation that is of greatest significance but rather the magnitude. Within the community of ‘Rubiscologists’, the enormous pile of proposals that have died on the reviewer’s sword – because of the idea that the trade-off between speed and affinity is narrow and immutable – is often lamented. Many have argued that the tight relationship was caused more by a sampling bias than a fundamental constraint, and the data of Young *et al.* (2016) now show they are correct. Their data also show that diversity in subcellular environment will be as or more important than diversity in Rubisco sequence when selecting organisms whose Rubiscos can shed light on the catalytic mechanism.

‘Absence of evidence is not evidence of absence’: the old cliché highlights the largest problem facing those trying to improve plant productivity through the manipulation of Rubisco catalysis. The desire for a simple solution, i.e. the single magic catalytic parameter that everyone can measure, and an irrational fear of exploratory research efforts (oft derided as fishing trips) has severely impeded advancement of our understanding of Rubisco catalysis for over 30 years. This has had the odd effect of stifling the generation of new data despite an ever-increasing interest in the small amount of data available (Box 2). Early surveys of Rubisco catalytic properties from photosynthetic organisms (see references in Badger *et al.*, 1998) received a great deal of interest, but were conducted prior to our current knowledge about the diversity of Rubisco sequences and modern methods for accurately measuring catalysis. By focusing on the evolutionary line leading to land plants, they over-looked the Red Form I enzymes found in the highly diverse non-green algae. This led to the assumption that the most useful Rubiscos would be found in land plants, and this idea has crept into scientific dogma. Even when later studies were published showing that Red Form I Rubisco from non-green algae were different and potentially better, little attention was paid.

**Box 2. Demand for kinetic data is outstripping supply**

Interest in Rubisco kinetics is ever increasing yet the number of papers forming the canon (containing the words ribulose, carboxylase and kinetic* in Web of Science^TM^) peaked two to three decades ago. A new peak in data is overdue.

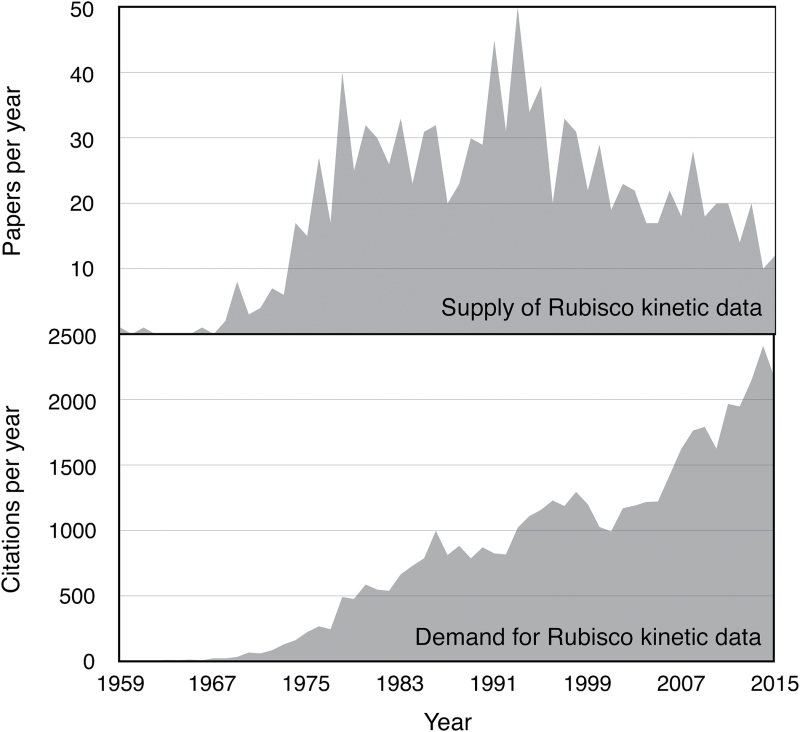

## Looking forward

Roughly half a century after discovering the importance of Rubisco in photosynthesis and photorespiration, kinetic properties have only been comprehensively studied for three Form I Rubiscos – tobacco, spinach and the cyanobacterium *Synechococcus* – and even those could benefit from more work. Young *et al.* (2016) demonstrate the need for studying Rubisco from a greater diversity of species and the value of measuring Rubisco kinetic parameters for both CO_2_ and O_2_ catalysis. However, to make a truly quantum leap in our understanding, a wider range of parameters should be measured. In addition to *k*_cat_ and *K*_m_ for CO_2_ and O_2_, and specificity for CO_2_ over O_2_, it is essential to include assays of the *K*_m_ for the other substrate, RuBP, along with binding kinetics of other known inhibitors and activators, frequency and forms of catalytic misfires, and ideally fractionation between isotopologues of CO_2_ and O_2_. These assays should be conducted using different combinations of large and small subunits and paired with measures of activation by multiple forms of Rubisco activase. Finally, given the paramount role of environment it will be critical to conduct assays across biologically relevant temperatures along with other subcellular properties like changes in molecular crowding. Now the chains are off and the kinetics research can get moving again.
